# Legionnaires’ Disease in Occupational Settings: A Cross-Sectional Study from Northeastern Italy (2019)

**DOI:** 10.3390/tropicalmed8070364

**Published:** 2023-07-16

**Authors:** Matteo Riccò, Pietro Ferraro, Silvia Ranzieri, Giorgia Boldini, Ilaria Zanella, Federico Marchesi

**Affiliations:** 1 Servizio di Prevenzione e Sicurezza Negli Ambienti di Lavoro (SPSAL), AUSL-IRCCS di Reggio Emilia, Via Amendola n.2, I-42122 Reggio Emilia, Italy; 2 Occupational Medicine Unit, Direzione Sanità, Italian Railways’ Infrastructure Division, RFI SpA, I-00161 Rome, Italy; dott.pietro.ferraro@gmail.com; 3 Department of Medicine and Surgery, University of Parma, Via Gramsci, 14, I-43126 Parma, Italy; silvia.ranzieri@unipr.it (S.R.); gboldini@ausl.pr.it (G.B.); ilaria.zanella@unipr.it (I.Z.); federico.marchesi@unipr.it (F.M.); 4 Servizio di Igiene Pubblica, AUSL di Parma, Via Vasari n.13/a, I-43123 Parma, Italy

**Keywords:** *Legionella pneumophila*, Legionnaires’ disease, occupational exposure, diagnosis, epidemiology, risk assessment

## Abstract

In Italy, Legionnaires’ Disease (LD) causes >1000 hospital admissions per year, with a lethality rate of 5 to 10%. Occupational exposures could reasonably explain a substantial share of total cases, but the role of Occupational Physicians (OPs) in management and prevention of LD has been scarcely investigated. The present survey therefore evaluates the knowledge, attitudes and practices (KAP) regarding LD from a convenience sample of Italian OPs, focusing on their participation in preventive interventions. A total of 165 OPs were recruited through a training event (Parma, Northeastern Italy, 2019), and completed a specifically designed structured questionnaire. The association between reported participation in preventive interventions and individual factors was analyzed using a binary logistic regression model, calculating corresponding multivariable Odds Ratio (aOR). Overall, participants exhibited satisfactory knowledge of the clinical and diagnostic aspects of LD, while substantial uncertainties were associated epidemiological factors (i.e., notification rate and lethality). Although the majority of participating OPs reportedly assisted at least one hospital (26.7%) and/or a nursing home (42.4%) and/or a wastewater treatment plant, only 41.8% reportedly contributed to the risk assessment for LD and 18.8% promoted specifically designed preventive measures. Working as OPs in nursing homes (aOR 8.732; 95% Confidence Intervals [95%CI] 2.991 to 25.487) and wastewater treatment plants (aOR 8.710; 95%CI 2.844 to 26.668) was associated with participation in the risk assessment for LD, while the promotion of preventive practice was associated with working as an OP in hospitals (aOR 6.792; 95%CI 2.026 to 22.764) and wastewater treatment plants (aOR 4.464, 95%CI 1.363 to 14.619). In other words, the effective participation of the OP in the implementation of preventive measures appears uncommon and is limited to certain occupational settings. Collectively, these results highlight the importance of tailoring specifically designed information campaigns aimed to raise the involvement of OPs in the prevention of LD in occupational settings other than healthcare.

## 1. Introduction

Legionellosis can be defined as the clinical syndrome resulting from an infection caused by bacteria belonging to the genus *Legionella* [1,2,3], which are accountable for two different clinical presentations, Pontiac Fever (PF) and Legionnaires’ Disease (LD) [2,3]. The first has been defined as a self-limiting febrile flu-like syndrome, while LD is a severe pneumonia that can be complicated by significant extra-pulmonary manifestations, such as renal failure or pericarditis [2,3]. To date, genus *Legionella* encompasses a total of 60 species and almost 70 distinct serogroups. Even though most human cases in western countries have been associated with the *L. Pneumophila* serogroup 1 (Lp1) [4], the share of cases associated with the non-Lp1 serogroup has recently increased [1,2,3,5,6], especially in Australia and New Zealand [7]. As regards epidemiology, there is considerable variability in overall prevalence data among different geographic areas, reflecting the use of different diagnostic tests (PCR, culture, or urine antigen test), the efficiency of the surveillance systems, and climate and/or other geographically linked risk factors [1,3,8]. This approximation makes actual incidence data unknown; however, notification systems showed an increasing incidence during the last decade for North America, the European Union/European Economic Area (EU/EEA) [1,3,8], and particularly Italy [9,10]. As recently summarized, Italian age-adjusted incidence rates for LD have increased from 1.053 cases per 100,000 in 2004 to 4.559 per 100,000 in 2019 [9], and despite a certain reduction in notification rates during the first year of the pandemic [10], crude incidence estimates for 2022 show an increase to 5.19 cases per 100,000 [11].

Susceptible people (that is, subjects aged > 50 years, of male sex, with history of chronic lung diseases, diabetes, and immunodeficiency conditions) usually become infected through inhalation of aerosols or aspiration of contaminated water [1,2,3,12,13,14], since these bacteria are ubiquitous in aquatic environments, both artificial and natural, where they can infect their natural hosts (i.e., amoeba and protozoa, primarily from genus *Acanthamoeba* and *Naegleria*) [3]. This way, any source of water, soil, compost, stagnant fluids, and ultimately artificial systems producing steam or emitting wet vapors should be considered as potentially contaminated by *Legionella* spp. [8,15,16,17,18]. All environmental factors that contribute to the exposure of susceptible people to sources of Legionella can be therefore acknowledged as key in explaining the epidemiology of LD and PF; these mainly include travel, residence in a health-care facility, and proximity to steam or nebulized water sources [2,3,9,10,12,13,14,19]. As a consequence, based on the settings where the infection is identified, a single case of *Legionella* infection may be labeled as Community-Acquired LD (CALD), Travel-Associated LD (TALD), or Hospital-Acquired LD (HALD).

*Legionella* infections should also be considered as a remarkable public health issue in occupational settings [20,21,22,23,24,25]. As inter-human transmission of LD is unusual [1], it is likely that occupational exposure to competent reservoirs for *Legionella* could result in a substantial share of total reported cases [20]. Work-related LD infections are not properly accounted for but could reasonably occur in many different situations, such as travelling, working with water plants or wet vapor generators (e.g., plumbers, air-conditioning and cooling tower installers and technicians, wellness center and pool workers, gardeners, irrigation plant installers, wastewater workers, dentists, etc.), and working in healthcare facilities such as hospitals or nursing homes [20,21]. Moreover, the working population is growing older, and nowadays many workers report chronic diseases or immunodeficiencies due to drugs or other medical conditions in their pathological history, thus being at increased risk of developing severe LD if exposed to the pathogen in occupational settings [9,20,26]. Not coincidentally, a growing base of evidence suggests that a large share of cases could be considered work related [20,21,22,23].

Starting from these assumptions, we decided to investigate the state of the art of Italian Occupational Physicians (OPs’) knowledge about this topic. Where implemented by local legal frameworks, OPs are key players in health promotion in the workplace [27,28], being also involved in the communication of risk and participating in information networks for workers [29]. Moreover, Italian Occupational Health and Safety Legislation requires OPs to participate in the adaptation of workplaces in accordance with the requirements of workers and to inform workers about the pros and cons of recommended interventions [28,30]. Therefore, raising their awareness of LD could improve the surveillance system of this disease and increase the quality of current epidemiological data. On the other hand, a better understanding of LD among OPs could also improve risk communication and management in everyday practice, representing the first step of an integrated preventive strategy.

The present study was therefore designed in order to address the following hypotheses:

(a) Italian OPs have some familiarity with the management of LD because of their role in occupational settings;
(b) Italian OPs have a sufficient understanding of LD in order to provide appropriate contributions to risk assessment and risk management in occupational settings;
(c) Italian OPs are involved in the design and implementation of appropriate preventive measures for LD.

## 2. Materials and Methods

### 2.1. Study Design

A preliminary cross-sectional questionnaire study was designed according to the STROBE guidelines (see checklist in Table A1) and involved OPs operating in the Province of Parma (Northeastern Italy, Emilia Romagna Region). Participants were asked about their KAP towards *Legionella* spp. as well as LD and its prevention.

### 2.2. Study Population

A convenience sampling was collected among OPs participating in a continuous medical education (CME) seminar on Legionnaires’ disease in occupational settings that took place in Parma (Emilia Romagna, Northeastern Italy) in September 2019. Before the inception of the seminar, all participating OPs (210) were asked whether they would agree to participate in the present survey. The questionnaire was delivered before the seminar, and all participants had around 20 min to complete the questionnaire. For practical reasons, the questionnaire was then collected by hand at the end of the meeting, only from subjects who expressed consent for study participation. The addressed sample represented around one third of all OPs operating in the Emilia Romagna Region in September 2019 (594) and around 2.6% of all licensed Italian professionals (7958). As there was no previous study on the KAP of Italian medical professionals towards *Legionella* spp., no preventive sample size calculation was performed.

### 2.3. Ethical Considerations

The first page of the questionnaire included an informed consent detailing the aims of the study and explaining that all information would be gathered anonymously, handled confidentially, and stored only for the time needed for a collective and anonymous analysis (translation of the informed consent is provided in Table A2). OPs were informed that participation was strictly voluntary, that the study was conducted according to the guidelines of the Declaration of Helsinki, and that participating in the survey had no consequence on the completion of CME program. The questionnaire was designed in order to avoid the potential identification of participants based on the presented material. In other words, this study caused no plausible harm or stigma to participating individuals. Because of its anonymous nature, observational design, and lack of clinical data about patients that could configure the present research as a clinical trial, a preliminary evaluation by an Ethical Committee was not required, according to Italian law (Gazzetta Ufficiale No. 76, dated 31 March 2008; Supplementary File S1).

### 2.4. Questionnaire

The questionnaire was specifically designed based on the model of Zingg and Siegrist in their KAP studies about OPs and their preventive interventions on infectious diseases [27,31]. Its test–retest reliability was preventively assessed through a survey of 20 medical professionals completing the questionnaire at two different points in time. The beta-testing questionnaires were ultimately excluded from the final analyses. The questionnaire shared with participants did include the following areas of inquiry (see Table A3):

(1) Demographic data: Age, gender, seniority as OP, and whether the respondents practiced as OPs in hospitals, nursing homes, or wastewater treatment plants.
(2) Knowledge Status: Participants received specifically designed knowledge tests, including (a) 13 true/false statements (e.g., “LD typically has inter-human spreading”; FALSE) and (b) 2 multiple-choice questions. Every correct answer added +1 to a summary General Knowledge Score (GKS), while wrong indications or a missing/”don’t know” answer added 0 (potential range of GKS: 0 to 15). The internal consistency of the questionnaire (i.e., the degree of homogeneity among the included items) was assessed through the Cronbach alpha test. In general, a score ≥ 0.7 is considered acceptable [32]. Participants were then asked to report the perceived occurrence of a series of clinical signs and symptoms among the individual features of LD cases (range: “never”, i.e., unlikely to be noticed, to “always”, i.e., a consistent feature of the syndrome). Finally, a series of individual and environmental risk factors was shown to the participants, and they were asked whether they had any knowledge of their role in LD (“yes” vs. “no”).
(3) Risk perception: According to the definition provided by Yates, risk perception can be defined as the function of the perceived probability of an event and its expected consequences [33]. Therefore, following the model developed by Betsch and Wicker [27], a sum score (Risk Perception Score, RPS) was calculated as follows. Participants were initially requested to rate, through a 5-point Likert scale, the perceived frequency (F; “extremely infrequent”, score = 1; “infrequent”, score = 2; “neutral”, score = 3; “frequent”, score = 4; “very frequent”, score = 5) and the perceived severity (C; “not at all severe”, score = 1; “low severity”, score = 2; “neutral”, score = 3; “severe”, score = 4; “very severe”, score = 5) of LD in occupational settings. RPS (potential range 1 to 25) was then calculated as
  RPS = F × S(1)
(4) Attitudes: To begin with, participants were asked to rate whether they were confident in properly recognizing any case of LD. Respondents were then requested to rate, through a full Likert scale of 1 to 5, a series of diagnostic options (bronchoalveolar lavage, BAL; urinary antigen assay; chest computed tomography; chest X ray; clinical examination; range: “totally unsatisfying” to “totally satisfying”).
(5) Practices: Participants were requested to report whether they had previously managed any case of LD infection among assisted workers in the previous 5 years (yes vs. no) or whether any of their friends/relatives had been previously affected by LD (yes vs. no). Moreover, they were asked whether they had participated or not in the risk assessment for LD in any of the occupational settings in which they work (yes vs. no) and whether they had actively promoted any preventive measure for LD (yes vs. no).

### 2.5. Data Analysis

In a preliminary stage, both GKS and RPS were normalized to percent values, then dichotomized by median value in high vs. low estimates. All Likert scales were similarly dichotomized (i.e., score 4 and 5 vs. 1 to 3).

Descriptive analysis of continuous variables was performed by calculation of average ± SD. All continuous variables were initially tested for their distribution by means of the D’Agostino and Pearson omnibus normality test and by assuming a cut-off value equal to *p* < 0.100 for rejecting normal distribution. Normally distributed continuous variables were compared by means of Student’s t-test or ANOVA, where appropriate, while non-normally distributed continuous variables were compared through Mann–Whitney or Kruskal–Wallis tests for multiple independent samples. Similarly, the association between continuous variables was assessed through Pearson’s correlation coefficient (normally distributed variables) or Spearman’s rank correlation coefficient (non-normally distributed variables).

In order to ascertain the items of the knowledge test that were able to better discriminate between participants with “strong” and “weak” understanding of LD, we applied the approach suggested by Möltner and Jünger [33,34]: correlation of each item of the knowledge test with higher GKS (i.e., >median value) was initially assessed through Spearman correlation analysis; all questions with a rho > 0.3 were included in a Simplified Knowledge Score (SKS).

Descriptive analysis of categorical variables required the calculation of proportions and percent values, and their association with two outcome variables (i.e., “Participating in the risk assessment for LD” and “Promoting any preventive measure for LD”) was assessed through the chi-squared test. All categorical variables in the univariate analysis associated with higher risk perception and having a *p* value < 0.05 were included as explanatory variables in two distinctive models of binary logistic regression analysis in order to calculate their adjusted odds ratios (aORs) and respective 95% confidence intervals (95%CIs) (Model 1: factors associated with participation in the risk assessment for LD; Model 2: factors associated with the promotion of any preventive measure for LD).

All statistical analyses were performed by means of IBM SPSS Statistics 26.0 for Macintosh (IBM Corp. Armonk, NY, USA).

## 3. Results

Overall, 165 out of 210 potential participants completed the questionnaire and were included in the final sample (participation rate 78.6%), that is, 27.8% of OPs from the Emilia Romagna region and 2.1% of all licensed Italian OPs. Their characteristics are reported in Table 1.

### 3.1. Demographic Data

Briefly, the mean age of participants was 48.3 years ± 10.8 (30.3% were aged 50 years or more at the time of the survey), and 57.6% were males. Of them, 42.4% had personal expertise in the management of occupational medicine in nursing homes, 26.7% in hospital settings, and 26.7% in wastewater treatment plants. Seniority ≥ 10 years was reported by 87.3% of participants (mean seniority: 21.1 years ± 11.3).

### 3.2. Knowledge Status

**Figure A1:**
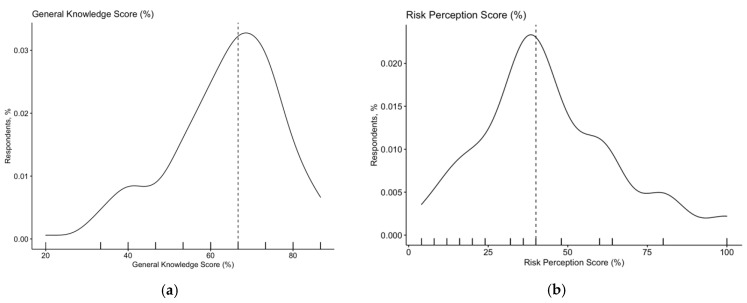
Summary of general knowledge test (**a**) and risk perception assessment (**b**). Distribution of General Knowledge Score (average: 63.7% ± 13.2; median = 66.7%; actual range: 20%–86.7%) was substantially skewed (D’Agostino Pearson, K2 11.47, *p* = 0.003) as was Risk Perception Score (average: 43.8% ± 21.4; actual range: 4 to 100%; D’Agostino Pearson, K2 = 10.40, *p* = 0.006).

The internal consistency coefficient amounted to Cronbach’s alpha = 0.796, with an acceptable reliability. After percent normalization, an unsatisfying GKS estimate of 63.7% ± 13.2 was calculated (median = 66.7%; actual range: 20% to 86.7%), with a total of 58 participants (35.2%) reporting a GKS > median value (“high knowledge status”). The sum score was substantially skewed, and its distribution did not pass the normality check (D’Agostino Pearson, K2 = 11.47, *p* = 0.003) (See Figure A1a). Detailed answers to the items of the knowledge test are reported in Table 2.

More precisely, statements associated with the clinical features of LD were associated with a high rate of correct answers (Q7, 95.3%; Q2, 81.5%; Q13, 80.2%; Q1, 79.2%); most identified LD as a non-vaccine preventable disease (Q7, 95.3%) and that mandatory notification to the local health unit is requested (Q11, 95.3%). In addition, statements based on the main microbiological features of Legionella pneumophila were associated with a high rate of correct answers (Q4, 79.2%; Q9 and Q5, both 71.7%; Q8, 67.9%; Q3, Q6, and Q10, all 63.2%). Substantial uncertainties were associated with three items: only 51.9% had knowledge international authorities should be notified of LD (Q12), while the most significant uncertainties were associated with epidemiological features. A similar share of respondents knew that every year around 1000 cases of LD are reported to Italian Health Authorities (Q15), while the actual case fatality rate (between 5% and 10%) was correctly identified by 26.4% of all respondents (Q14).

**Figure A2:**
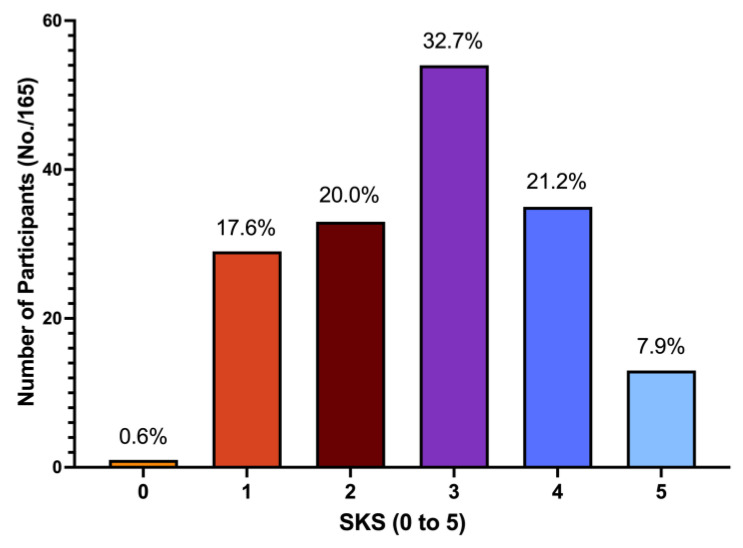
Summary of Simplified Knowledge Score (SKS), including all items from the GKS that were correlated with Higher Knowledge Status (GKS > median value) with a rho ≥ 0.300. With a potential range 0 to 5, 29.1% of participants scored either 4/5 or 5/5 correct answers.

Five items passed the cut-off value of rho > 0.3 and were included in the SKS: Q4 (rho = 0.357), Q6 (rho = 0.443), Q8 (rho = 0.301), Q12 (rho = 0.393), and Q15 (rho = 0.394). The SKS score potentially ranged from 0 to 5, and was arbitrarily dichotomized as score 0 to 3 (117 out of 165 participants, 70.9%) vs. score 4 to 5 (48/165, 29.1%) (Figure A2).

**Figure 1:**
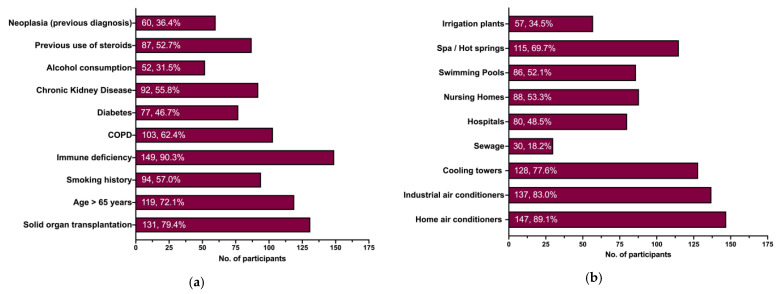
(**a**) Clinical risk factors for Legionnaires’ Disease (LD); (**b**) environmental risk factors for developing Legionella infections. Note: COPD = chronic obstructive pulmonary disease.

When dealing with the clinical risk factors for LD (Figure 1a), the majority of respondents identified immune deficiency (90.3%), followed by being a recipient of solid organ transplantation (79.4%), being aged 65 years or more (72.1%), being affected by COPD (62.4%), reporting a smoking history (57.0%), a history of chronic kidney disease (55.8%), or the previous use of steroids (52.7%). On the contrary, the previous diagnosis of diabetes, neoplasia, and the history of alcohol consumption were reported by less than half of participants (46.7%, 36.4%, and 31.5%, respectively).

As shown in Figure 1b, the most frequently reported environmental risk factor for LD was identified as the exposure to home air conditioners (89.1%), followed by industrial air coolers (83.0%) and cooling towers (77.6%). Around two thirds of participants similarly reported the exposure to spa and/or hot springs (69.7%), while swimming pools, nursing homes, and hospitals were acknowledged as environmental risk factors by around half of respondents (52.1%, 53.3%, and 48.5%, respectively). Irrigation plants (34.5%) and sewage systems (18.2%) were the less frequently reported items.

**Figure A3:**
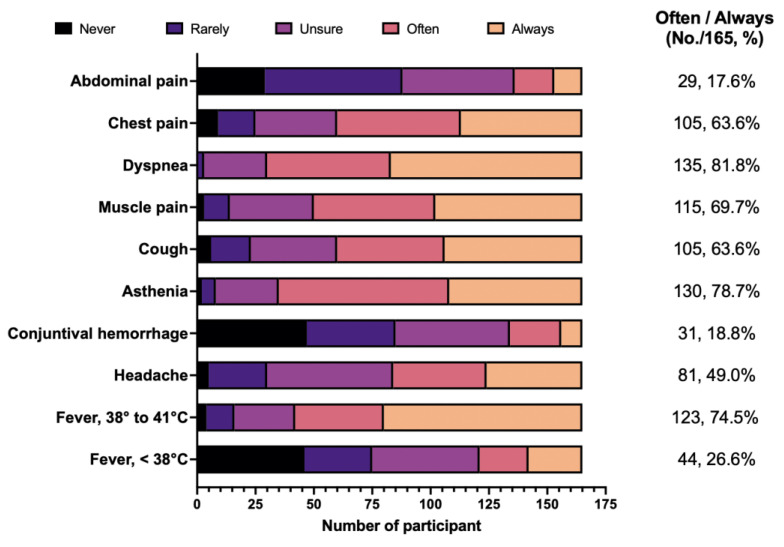
Perceived frequency of the occurrence of a series of selected clinical features of Legionnaires’ Disease as reported by 165 occupational physicians participating in the survey (Italy, 2019).

Regarding the clinical features considered as either often or always associated with LD, the most frequently reported one was dyspnea (81.8%), followed by asthenia (78.7%), fever of 38 to 41° C or muscle pain (69.7%), and cough and chest pain (63.6%), while symptoms such as headache, fever < 38 °C, conjunctival hemorrhage, and abdominal pain were reported by less than half of participants (49.0%, 26.6%, 18.8%, and 17.6%, respectively) (Figure A3).

### 3.3. Risk Perception

Overall, only 16.7% of participants acknowledged LD as a frequent issue in occupational medicine, while 76.4% identified LD as a severe condition; the cumulative was 43.8% ± 21.4 (actual range: 4 to 100%). The summary score was substantially skewed, as shown by the D’Agostino Pearson test (K2 = 10.40, *p* = 0.006) (Figure A1b).

### 3.4. Practices

When dealing with practices associated with LD, 41.8% of participating OPs had reportedly participated in the risk assessment of LD, while only 18.8% had promoted any preventive measure for LD. Overall, 32.7% of participants had any previous professional experience, while 27.9% had knowledge of at least one case of LD among assisted workers and 9.1% among friends or relatives.

### 3.5. Attitudes

**Figure A4:**
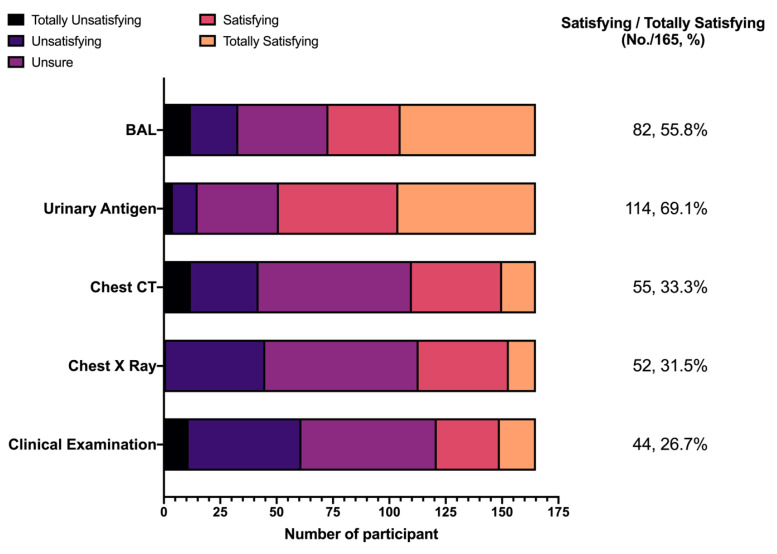
Confidence in diagnostic options for Legionnaires’ Disease among 165 occupational physicians participating in the survey (Italy, 2019).

Overall, 39.1% of medical professionals participating in the present survey were confident in their ability to properly diagnose LD cases. Around 69.1% considered urinary antigen testing to either satisfy or totally satisfy requirements for diagnosis, followed by BAL (55.8%), Chest CT scans (33.3%), Chest X rays (31.5%), and clinical examination (26.7%) (Figure A4).

### 3.6. Univariate Analysis

**Figure A5:**
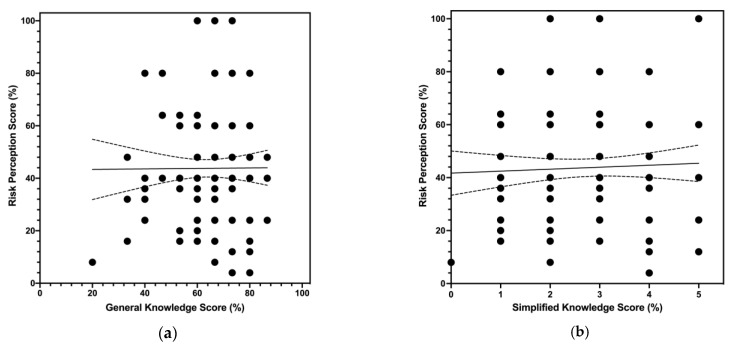
Correlation between: (**a**) Risk Perception Score and General Knowledge Score (Spearman’s rank correlation test, rho = 0.010, *p* = 0.897); (**b**) Risk Perception Score and Simplified Knowledge Score (Spearman’s rank correlation test, rho = 0.045, *p* = 0.568).

**Figure A6:**
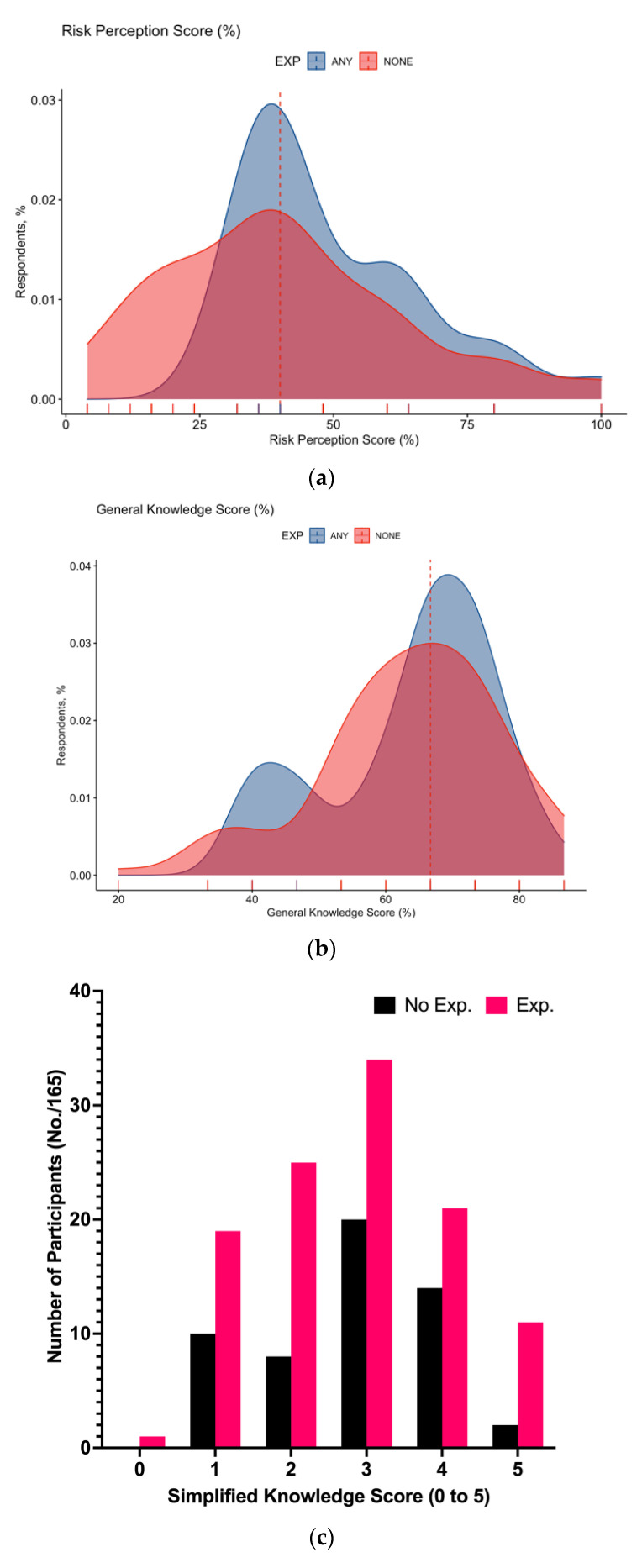
Comparison of summary scores by having reported any previous experience with Legionnaires’ disease or not. Regarding the General Knowledge Score (GKS), no substantial differences were identified between respondents having any (64.0% ± 12.6) or no previous experience (EXP) with LD (63.6% ± 14.5; Mann–Whitney U = 3096.5, *p* = 0.726) (**a**). Comparison of Risk Perception Score by having or not having any previous experience with Legionnaires’ Disease; participants reporting any previous experience with LD (50.0% ± 17.6) reported a substantially higher score than those reporting no previous interaction with the disease (40.8% ± 22.5; Mann–Whitney U = 3820.0, *p* = 0.004) (**b**). Simplified Knowledge Score did not show substantial differences between participants having or not having any previous experience with LD (2.81 ± 1.13 vs. 2.79 ± 1.24; U = 3069.0, *p* = 0.797) (**c**).

As shown in Table 3, no correlation was found between knowledge status and risk perception (Spearman’s rank correlation test: rho = 0.010, *p* = 0.897 for RPS vs. GKS, and rho = 0.045, *p* = 0.568 for RPS vs. SKS) (see Figure A5). When knowledge status reported by participating OPs was compared for those having reported any previous experience with LD or not, no substantial differences were identified between the former OPs and those having no previous experience with LD, both for GKS (64.0% ± 12.6 vs. 63.6% ± 14.5; Mann Whitney U = 3096.5, *p* = 0.726) and for SKS (2.81 ± 1.13 vs. 2.79 ± 1.24; U = 3069.0, *p* = 0.797). On the other hand, in the comparison of RPS in those having or not having any previous experience with Legionnaires’ disease, participants reporting any previous experience with LD (50.0% ± 17.6) had a substantially higher score than those reporting no previous experience (40.8% ± 22.5; Mann Whitney U = 3820.0, *p* = 0.004) (See Figure A6).

In bivariate analysis, having previously participated in the risk assessment of LD was associated with actively promoting any preventive measure for LD (30.4% vs. 10.4%, *p* = 0.002), having had any previous practice as OPs in hospital settings (50.7% vs. 9.9% of those having not participated in the risk assessment; *p* < 0.001), nursing homes (73.9% vs. 19.8%, *p* < 0.001), and wastewater treatment plants (52.2% vs. 8.3%, *p* < 0.001). Interestingly, OPs having participated in the risk assessment for LD were associated with high SKS (40.6% vs. 20.8%, *p* = 0.010).

By taking the previous promotion of any preventive measure for LD as the outcome variable, a positive association was found with having participated in the risk assessment for LD (67.7% vs. 35.8%, *p* = 0.002). Moreover, a positive association was also found with male gender (83.9% vs. 51.5%, *p* = 0.002), and with having practiced occupational medicine in hospital settings (61.3% vs. 18.7%, *p* < 0.001), nursing homes (67.7% vs. 36.6%, *p* = 0.003), and wastewater treatment plants (54.8% vs. 20.1%, *p* < 0.001).

### 3.7. Multivariable Analysis

As shown in Table 4, multivariable regression models included the following variables:

Model 1 (participating in the risk assessment for LD): reporting any practice as an OP in hospital settings, nursing homes, wastewater treatment plants; promoting any preventive measure for LD; reporting high knowledge status.

Model 2 (promoting preventive measures for LD): reporting any practice as an OP in hospital settings, nursing homes, wastewater treatment plants; participating in the risk assessment for LD.

In Model 1, the outcome variable was positively associated with the status of working as an OP in nursing homes (aOR 8.732; 95%CI 2.991 to 25.487) and wastewater treatment plants (aOR 8.710; 95%CI 2.844 to 26.668). In Model 2, the outcome variable was positively associated with reporting any practice as an OP in hospitals (aOR 6.792; 95%CI 2.026 to 22.764) and wastewater treatment plants (aOR 4.464, 95%CI 1.363 to 14.619).

## 4. Discussion

Here we report on a cross-sectional study of 165 Italian OPs shortly before the inception of the SARS-CoV-2 pandemic about their understanding of LD in occupational settings. We specifically addressed the following endpoints: (a) whether Italian OPs have any familiarity with LD; (b) whether Italian OPs have a sufficient understanding of LD in order to provide appropriate contributions to risk assessment and risk management in occupational settings; (c) whether Italian OPs are involved or not in the design and implementation of appropriate preventive measures for LD in workplaces.

*Familiarity of Italian OP with LD*. Participating OPs exhibited a substantial lack of familiarity with the topic of LD, as only one third of all participants had any previous experience with disorders associated with *Legionella* spp., including LD and PD cases diagnosed among assisted workers and friends/relatives. Less than 40% of medical professionals were confident in their ability to properly recognize incident cases of LD. Overall, these results were quite unexpected. The occurrence of LD in Italy has steadily increased from the early 2000s (age-adjusted incidence rate, AIR: 1.053/100,000 people, 95%CI 0.896 to 1.237) to 2019 (4.669/100,000, 95%CI 4.251 to 5.088 [9,10]), with more than 1500 officially reported cases since 2015 [7,9,19,34,35]. Within this timeframe, cases of HALD have progressively decreased [9,11], benefiting from appropriate preventive interventions from regulatory agencies [11,36,37]. Between 2004 and 2019, only 5.9% of cases were associated with healthcare settings, including 523 cases from residential homes [9]. The growing share of community-acquired LD (around 85% of incident cases in 2023) suggests that most may be misclassified, being reasonably work related [11,38]. Indeed, as recently summarized [9], even though the majority of Italian cases of LD are usually identified in individuals from older age groups (i.e., between 2004 and 2019, 62.7% of officially reported cases were in those aged ≥ 60 years), around 37% conversely occurred in individuals of working age. Moreover, Italian reports on LD between 2002 and 2011 did provide some insights into the occupations of the reported cases [39,40], and a potential link with the occupational settings was thought to range between 11% (2009 and 2011) and 21% (2005). Not coincidentally, since 2007, the European Agency for Safety and Health at Work (EU-OSHA) has acknowledged *Legionella* as a significant biological risk agent in occupational settings [20], as recently summarized by Principe et al. [20] and Petti and Vitali [23]. In other words, while real world data would suggest the ever-increasing occurrence of LD for OPs, our survey highlights that infections from *Legionella* spp. are still perceived as a marginal issue.

*Knowledge status of participating OPs*. Participating OPs exhibited an unsatisfying knowledge status. With a potential range of 0 to 100%, the actual GKS was estimated to be 63.7% ± 13.2, and only 29.1% of participants exhibited a high understanding of LD, as summarized by the calculation of SKS. SKS was designed in order to only include items that were characterized as “stronger” effectors of the actual knowledge status [41,42], more precisely: the temperature associated with a better growth of Legionella pneumophila (Q4), the capability of the same *Legionella* strain to cause both PF and LD (Q6), the length of clinical incubation for LD (Q8), the requirement for international notification of incident cases (Q12), and the actual notification rate (Q15) [8,15,16,17,43].

In fact, a substantial share of participating professionals exhibited a relatively satisfying understanding of the specific physical conditions required for proliferation of *Legionella* spp. and were also able to properly characterize their common reservoirs (i.e., cooling towers and air-conditioning systems), with the notable exception of sewage and irrigation plants. In the systematic review of Principe et al. [20], irrigation plants and sewage treatment plants were identified as high-risk settings for LD. As a consequence, the knowledge of participating OPs regarding this information was lacking, particularly when compared to the share of respondents who identified working in healthcare settings and more precisely in retirement and/or nursing homes as risk factors for LD (around 50% for both claims), seemingly ignoring the recent trend from official reports [6,9,11,19,35,40,44]. Moreover, several studies have assessed seroprevalence rates and actual risk factors for *Legionella* among the staff of residential homes and hospitals, and their results collectively point towards non-healthcare-related sources of primary infection [22,45]. Noticeable knowledge gaps were also associated with the actual epidemiology of LD: less than 20% of respondents were able to identify the actual notification rate for LD (over 1000 cases/year) [9,10], while only one quarter of participating OPs correctly reported the case fatality ratio of around 5.0% for the time period 2004–2019 (Q14) [9,10]. Respondents also showed a lack of awareness regarding their potential requirements after a diagnosis of LD, as only 51.9% were aware of the international notification requirements for incident cases [46,47,48,49,50,51]. In other words, both factual and conceptual knowledge of participating OPs was lacking, particularly from a Public Health point of view [18,52,53]. Even though our study was not preventively designed in order to assess all the knowledge domains included in the revised Bloom’s taxonomy, results from knowledge test, particularly when focusing on SKS, suggest that participating OPs struggled in proficiently contributing to the appropriate implementation of preventive measures [54,55]. Several factors may contribute to these substantial knowledge gaps. First of all, OPs are the medical professionals involved in health surveillance and preventive interventions in workplaces [27,56,57,58,59], and pathogens such as *Legionella* are only cursorily addressed in the conventional core curriculum of Italian post-degree specialization courses in Occupational Medicine [60,61]. While only one third of participants had any previous interaction with LD, the majority exhibited a relatively good understanding of the clinical features of this disorder, including the pros and cons of diagnostic options. It is reasonable that OPs may have developed their understanding of LD from a perspective other than that of Occupational Medicine, perhaps through their clinical background, which is not necessarily based on up-to-date information. This point of view of OPs regarding the actual features of LD in occupational settings is supported by the reported risk factors. Even though current evidence has repetitively associated LD with individual factors such as older age, immune depression, diabetes, and pre-existing respiratory diseases [2,9,13,47,62], when reported, risk factors for infected workers were limited to male gender (149/178 cases, 83.7%), smoking (47/102 cases; 46.1%), alcohol consumption (1/43 cases; 2.3%), and evidence of previous poor health conditions (23/102 cases; 22.5%) [20]. In other words, it is reasonable that, even in this case, the answers from participating OPs did not reflect the actual expertise of Occupational Medicine professionals, but were rather associated with their original clinical background.

*Participation of OPs in the prevention of LD in occupational settings*. Only 41.8% of participating professionals reportedly contributed to the risk assessment of LD in occupational settings, and 18.8% had promoted any preventive measure for LD. We tentatively assessed the main effectors for participating in the risk assessment and promotion of preventive interventions, and all of them were associated with professional factors, while knowledge status and risk perception did not have an association with either outcome variable. In fact, OPs working in healthcare facilities (such as hospitals and nursing homes) and wastewater treatment plants were more likely to participate in the risk assessment and promotion of preventive measures. Current Italian guidelines for the prevention of LD may have contributed these results [37]. According to up-to-date recommendations, medical professionals should be actively involved in the risk assessment for LD and in the design of preventive interventions, particularly in settings usually acknowledged to have high risk, more precisely: healthcare settings, residential homes, wastewater treatment plants, and swimming pools/spas. In such settings, and particularly for small and middle- sized enterprises, OPs could be the sole medical professionals reporting a background in preventive medicine and public health and thus be actively involved in the risk assessment for LD [60,63,64]. In other words, while OPs should actively contribute to the prevention of LD by primarily targeting occupational health and safety [20,21,22,23,24,25,65], they are reasonably requested to promote interventions focused on the general population and/or hosts and/or residents of healthcare or travel settings, eventually developing a radically biased point of view. On the other hand, we could speculate that OPs having contributed to risk assessment for LD of parent employers may have developed a greater attention to this topic, leading to their commitment to LD management and prevention, even in settings other than those where conventional legal frameworks would require their intervention.

**Figure A7:**
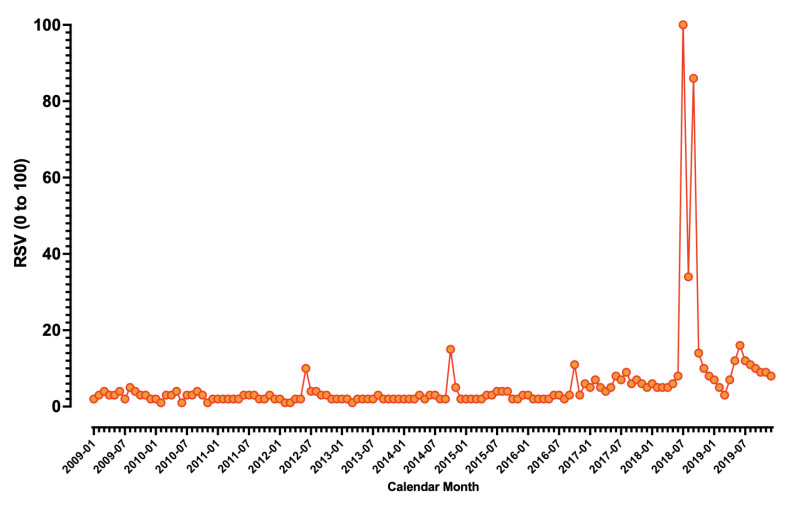
Evolution over time (January 2009 to December 2019) of the relative search volumes (RSV, value 0 to 100) for Legionnaires Disease in Italy. RSV is the ratio between the queries over a specific term (in this case, Legionnaires’ disease) and the total web queries in that specific timeframe.

Another explanation of the participation of OPs in the preventive measures for LD may be found in the relatively low estimates for risk perception. The very low share of participants acknowledging the high frequency of LD in occupational settings not only reasonably reflects the common knowledge framework on LD [20], but could represent a potential consequence of the information otherwise conveyed by new and conventional media, a particularly critical issue when dealing with the knowledge status and risk perception of OPs [58,64,66,67,68,69,70]. *Legionella* infections have repetitively evoked both media and public interest, which in turn has resulted in high pressure on medical professionals. As media interest is usually focused on outbreaks occurring in healthcare settings such as nursing homes—where the fatality rates are usually higher than in community-acquired cases [20]—media coverage has reasonably contributed to the improper understanding of the actual occupational risk factors for LD. In this regard, internet search data can provide some reliable proxy for the interest on LD conveyed over time by new media. In particular, calculation of relative search volumes (RSVs) through Google Trends^TM^ (the open online tool specifically developed by Google^TM^) has been shown as quite effective in reporting interest in a specific keyword or search topic in terms of performed queries, particularly when dealing with infectious diseases [71,72,73]. As shown in Figure A7, a peak of web searches was identified during the second half of 2018, during an unprecedented outbreak of LD associated with serogroup 2 [6], while the overall estimates appeared substantially low before and immediately after, stressing the general underestimation of and scarce interest in this disorder.

*Limits*. Despite its novelty, and the potential significance for the daily practice of OPs, the present study is unfortunately affected by several substantial limitations that must be addressed and discussed.

First of all, we must acknowledge some implicit limitations of our instrument. In this study, we deliberately focused the assessment of knowledge status on the dimension of factual knowledge, while other dimensions could contribute to the cognitive processes—at least, according to the revised Bloom’s taxonomy [54,55]. Still, it should be stressed that our study did not focus on individuals’ conventional training but rather targeted medical professionals regarding the active application of what they learned during their education. As a consequence, the questionnaire was not deliberately designed through the usual hierarchy of thinking skills (i.e., remember, understand, apply, analyze, evaluate, create). Using the Health Belief Model, our instrument was focused on the perceptions of disease susceptibility and severity, barriers to health practices, benefits, self-efficacy, and cues to action [74,75,76,77]. In fact, we postulated that participants’ belief in the health threat represented by LD, as well as the belief in the effectiveness of preventive intervention [78], would be modelled by individual experience and understanding of the assessed topic, representing the main predictors for the likelihood that the person adopted the assessed behavior (i.e., contributing to LD prevention) [79].

Second, our convenience sample was quite small. Despite a relatively high response rate (78.6% of all addressed participants), it only included 2.1% of all licensed Italian OPs. Nonetheless, we targeted a very specific topic, and to our knowledge, no previous KAP studies on LD in occupational settings have been previously performed [20,23]. Moreover, as the same disease burden could be difficult to ascertain, any preventive sample size calculation would be reasonably impaired by its roots. In order to cope with this specific shortcoming, we opted for a convenience sample including a relatively large number of professionals from a delimited geographic area; in fact, the sample encompassed 27.8% of all OPs from the Emilia Romagna region. Still, as Emilia Romagna is characterized among all Italian Regions by the highest notification rates [9], a certain overestimation of LD by the recruited participants cannot be ruled out. As a consequence, the generalizability of our results is hampered not only by the overall sample size, but also by the oversampling of professionals with a better understanding of the addressed topic. Likewise, by its design and the sampling strategy, our study is also reasonably affected by the extensive overrepresentation of participants exhibiting a pre-existing interest in LD [27,66,80]. Therefore, the baseline knowledge we specifically addressed could exceed that of OPs from other areas, also considering the distinctive training during the residency program in occupational medicine in Italian and international settings [60]. The potential sampling issues are also stressed by the demographic characteristics of participating professionals. While the Italian medical workforce is increasingly older, the mean age of the respondents was well below 50 years at the time of the survey, and only one third of participants were older than 50 years. These figures are substantially lower than national estimates for the medical workforce (around 55 years of age) [81] and collectively suggest the substantial oversampling of younger OPs [29,30,32].

Third, we cannot rule out the possibility that both GKS and RPS estimates may have been affected by some degree of social desirability bias, with participants reporting the answers that they understood as “socially appropriate” rather than their authentic ones [58]. In other words, not only were both estimates either partially or totally unsatisfying, but our quantitative assessment may have been biased, leading to even worse actual figures. As these issues are usually associated with KAP studies, in order to cope with these shortcomings, we implemented a flexible and reliable design for knowledge testing and risk perception assessment [27,74,82]; however, a cautionary approach to our results is required. In this regard, it should be stressed that the very same design of the knowledge test could be criticized, as it mostly relied on true–false items, also including two multiple choice items with a single best answer. As previously stressed by Krebs [83], this approach could be affected by reduced selectivity, potentially associated with a significant share of correct answers given by chance rather than by the actual knowledge status of the respondent. In order to overcome this potential shortcoming, future iterations of our questionnaire will not only prioritize “strong” predictors of knowledge status (i.e., Q4, Q6, Q8, Q12, and Q15) but will also include items requiring the correct judgement of all reported statements (type Kprime) in order to reduce the potential impact of items potentially affected by traditional flaws such as clueing and guessing.

Fourth, the collected data were self-reported and not externally validated. As a consequence, we are unable to ascertain the accuracy of the reported data (i.e., having or not participated in the risk assessment for LD and implementation of preventive measures). Similar shortcomings affect the reporting of potential KAP effectors such as having previously managed any LD case. As a considerable amount of evidence suggests that personal experiences of a certain health threat represent the main driver of the eventual KAP of OPs [27,67,74,75], we were unable to objectively ascertain the actual occupational background of recruited participants, which represents a substantial limit of the present study.

Last, the present study was performed before the inception of the SARS-CoV-2 pandemic. There is considerable evidence that the pandemic and associated non-pharmaceutical interventions had a substantial effect on the epidemiology of LD: during 2020, a substantial decrease in notification rates was reported in several European countries, particularly in Italy, and this trend also involved hospital-associated cases [10]. On the contrary, the SARS-CoV-2 pandemic was associated with a sustained increase in the actual case fatality ratio of nosocomial cases, particularly among co-infections [38,84,85,86,87]. Moreover, the pandemic increased general interest in air-conditioning systems as preventive measures for reducing the spread of airborne pathogens in closed environments [88,89,90]. Therefore, a cautious appraisal of our results should consider that a follow-up survey would presumptively report substantial differences in knowledge status and risk perception for LD.

## 5. Conclusions

Despite some significant limits in its design, our study suggests that shortly before the inception of the SARS-CoV-2 pandemic, only a limited share of OPs from Northeastern Italy participated in risk assessment and preventive interventions for LD in occupational settings. Moreover, OPs showed a significant underestimation of the occupational risk for LD. As the share of work-related LD remains improperly ascertained, but could peak to around one fifth all of incident cases, interventions aimed to specifically improve the professional expertise of OPs regarding this pathogen could improve their capability to share appropriate preventive interventions. Despite the limits of the present study, including a reduced sample size and the lack of external validation, our results could provide a benchmark for future studies monitoring the knowledge status of OPs on LD, more properly characterizing the preventive measures they promote and their commitment to guaranteeing safer and healthier workplaces.

## Figures and Tables

**Table 1:** Characteristics of 165 occupational physicians participating in the survey (Province of Parma, 2019) (Note: LD = Legionnaires’ Disease).

General Characteristics of the Sample	No./165, %	Average ± S.D.
Age		48.3 ± 10.8
≥50 years	50, 30.3%	
Gender (No., %)		
Male	95, 57.6%	
Female	64, 38.8%	
Undisclosed	6, 3.6%	
Seniority		21.1 ± 11.3
≥10 years (No., %)	144, 87.3%	
Any practice as Occupational Physician in		
Hospitals	44, 26.7%	
Nursing homes	70, 42.4%	
Wastewater treatment plants	44, 26.7%	
Participation in the risk assessment for LD	69, 41.8%	
Promotion of any preventive measure for LD	31, 18.8%	
Case of LD among assisted workers	46, 27.9%	
Case of LD among friends/relatives	15, 9.1%	
Previous experience with LD	54, 32.7%	
General Knowledge Score		63.7% ± 13.2
>median (66.7%)	58, 35.2%	
Simplified Knowledge Score 4 to 5	48, 29.1%	
Risk Perception		
Acknowledging LD as a frequent issue in occupational settings	27, 16.4%	
Acknowledging LD as a severe issue in occupational settings	126, 76.4%	
Risk Perception Score		43.8% ± 21.4
>median (40.0%)	66, 40.0%	
Confidence in being able to recognize an LD case	81, 39.1%	

**Table 2:** Knowledge test results of 165 occupational physicians participating in the survey (Province of Parma, 2019) (Notes: LD = Legionnaires’ Disease; (+) = item correlated with higher knowledge status, with rho ≥ 0.300).

Statement	Answer	No./165, %	Correlation with Higher GKS (rho)
Q1. LD typically has inter-human spreading	FALSE	84, 79.2%	−0.043
Q2. Immunocompromised patients are at higher risk for developing LD	TRUE	97, 81.5%	0.233
Q3. *Legionella pneumophila* is rare in the environment	FALSE	67, 63.2%	0.241
Q4. *Legionella pneumophila* optimal growth occurs between 32 and 40 °C	TRUE	84, 79.2%	0.357 (+)
Q5. *Legionella pneumophila* replicates between 20 and 50 °C	TRUE	76, 71.7%	0.231
Q6. *Legionella* strains able to cause Pontiac Fever cause Legionellosis	TRUE	67, 63.2%	0.443 (+)
Q7. Legionellosis is a vaccine-preventable disease	FALSE	101, 95.3%	−0.049
Q8. Incubation for legionellosis ranges between 2 and 10 days	TRUE	72, 67.9%	0.301 (+)
Q9. Macrolides and Quinolones can be used in cases of suspected LD	TRUE	76, 71.7%	0.243
Q10. LD occurs in less than 5% of all patients exposed to waters contaminated by *Legionella*	TRUE	67, 63.2%	0.291
Q11. LD must be officially reported to the Local Health Unit	TRUE	101, 95.3%	0.166
Q12. LD is a notifiable disease to international authorities	TRUE	55, 51.9%	0.393 (+)
Q13. LD follows ingestion of contaminated water	FALSE	85, 80.2%	0.053
Q14. Case fatality for Legionellosis is			0.114
<1%	FALSE	9, 8.5%	
between 1 and 5%	FALSE	38, 35.8%	
between 5 and 10%	TRUE	28, 26.4%	
between 10 and 15%	FALSE	21, 19.8%	
>15%	FALSE	10, 9.4%	
Q15. Every year … are reported in Italy			0.394 (+)
less than 100 cases	FALSE	13, 12.3%	
100 to 200 cases	FALSE	26, 24.5%	
200 to 500 cases	FALSE	21, 19.8%	
500 to 1000 cases	FALSE	13, 12.3%	
over 1000 cases	TRUE	21, 19.8%	

**Table 3:** Bivariate analysis of factors associated with the outcome variables of a) participating in the risk assessment for Legionnaires’ disease (LD) and b) promoting any preventive measure against Legionnaires’ disease (LD) for 165 Italian Occupational Physicians participating in the survey (2019).

General Characteristics of the Sample	Participation in the Risk Assessment for LD			Promotion of any Preventive Measures for LD		
	Ever (No./69, %)	Never (No./96, %)	Chi- Squared Test *p* Value	Ever (No./31, %)	Never (No./134, %)	Chi- Squared Test *p* Value
Age ≥ 50 years	24, 34.8%	26, 27.1%	0.374	10, 32.3%	40, 29.9%	0.963
Male Gender	36, 52.2%	59, 61.5%	0.303	26, 83.9%	69, 51.5%	0.002
Seniority ≥ 10 years	63, 91.3%	81, 84.4%	0.280	31, 100%	113, 84.3%	0.059
Any practice as Occupational Physician in						
Hospitals	35, 50.7%	9, 9.4%	< 0.001	19, 61.3%	25, 18.7%	< 0.001
Nursing homes	51, 73.9%	19.8%	< 0.001	21, 67.7%	49, 36.6%	0.003
Wastewater treatment plants	36, 52.2%	8, 8.3%	< 0.001	17, 54.8%	27, 20.1%	< 0.001
Case of LD among assisted workers	21, 30.4%	25, 26.0%	0.656	8, 25.8%	38, 28.4%	0.950
Case of LD among friends/relatives	3, 4.3%	12, 12.5%	0.128	0, -	15, 11.2%	0.108
Previous experience with LD	21, 30.4%	33, 34.4%	0.716	8, 25.8%	46, 34.3%	0.485
Participation in the risk assessment for LD	-	-	-	21, 67.7%	48, 35.8%	0.002
Promotion of preventive measures for LD	21, 30.4%	10, 10.4%	0.002	-	-	-
Simplified Knowledge Score 4 to 5	28, 40.6%	20, 20.8%	0.010	13, 41.9%	35, 26.1%	0.127
Risk Perception > median (40.0%)	30, 43.5%	36, 37.5%	0.540	13, 41.9%	53, 39.6%	0.968
Confidence in being able to recognize an LD case	35, 50.7%	46, 47.9%	0.843	13, 41.9%	68, 50.7%	0.493

**Table 4:** Regression analysis of factors associated with the outcome variables of a) participating in the risk assessment for Legionnaires’ disease (LD) and b) promoting any preventive measure against LD. Adjusted Odds Ratios (aOR) with respective 95% Confidence Intervals (95%CI) were calculated by means of binary logistic regression that included all variables that in bivariate analysis were associated with the outcome variable having a *p* < 0.05.

	Participation in the Risk Assessment for LD (aOR, 95%CI)	Promotion of any Preventive Measure for LD (aOR, 95%CI)
Practice as Occupational Physician in		
Hospitals	2.850 (0.936; 8.676)	6.792 (2.026; 22.764)
Nursing homes	8.732 (2.991;25.487)	0.902 (0.257; 3.164)
Wastewater treatment plants	8.710 (2.844; 26.668)	4.464 (1.363; 14.619)
Participation in the risk assessment for LD	-	1.368 (0.401; 4.663)
Promotion of any preventive measure for LD	1.495 (0.453; 4.929)	-
Simplified Knowledge Score 4 to 5	2.152 (0.847; 5.468)	-

## Data Availability

Data are available on request.

## Supplementary Materials

The following supporting information can be downloaded at: https://www.mdpi.com/article/10.3390/tropicalmed8070364/s1, Supplementary File S1: Translation of Gazzetta -Ufficiale no. 76, dated 31 March 2008.

## Appendix A

**Table A1:** STROBE Statement—Checklist of items that should be included in reports of *cross-sectional studies*.

	Item No.	Recommendation	Page
**Title and abstract**	1	(*a*) Indicate the study’s design with a commonly used term in the title or the abstract	1
		(*b*) Provide in the abstract an informative and balanced summary of what was done and what was found	1
**Introduction**			
Background/rationale	2	Explain the scientific background and rationale for the investigation being reported	1–2
Objectives	3	State specific objectives, including any prespecified hypotheses	2
**Methods**			
Study design	4	Present key elements of study design early in the paper	3
Setting	5	Describe the setting, locations, and relevant dates, including periods of recruitment, exposure, follow-up, and data collection	3
Participants	6	(*a*) Give the eligibility criteria, and the sources and methods of selection of participants	3–4
Variables	7	Clearly define all outcomes, exposures, predictors, potential confounders, and effect modifiers. Give diagnostic criteria, if applicable	3–4
Data sources/measurement	8	For each variable of interest, give sources of data and details of methods of assessment (measurement). Describe comparability of assessment methods if there is more than one group	4
Bias	9	Describe any efforts to address potential sources of bias	3–4
Study size	10	Explain how the study size was arrived at	3
Quantitative variables	11	Explain how quantitative variables were handled in the analyses. If applicable, describe which groupings were chosen and why	4
Statistical methods	12	(*a*) Describe all statistical methods, including those used to control for confounding	4
		(*b*) Describe any methods used to examine subgroups and interactions	4
		(*c*) Explain how missing data were addressed	4
		(*d*) If applicable, describe analytical methods taking account of sampling strategy	4
		(*e*) Describe any sensitivity analyses	-
**Results**			
Participants	13 *	(a) Report numbers of individuals at each stage of study—e.g., numbers potentially eligible, examined for eligibility, confirmed eligible, included in the study, completing follow-up, and analyzed	5
		(b) Give reasons for non-participation at each stage	5
		(c) Consider use of a flow diagram	5
Descriptive data	14 *	(a) Give characteristics of study participants (e.g., demographic, clinical, social) and information on exposures and potential confounders	5–6
		(b) Indicate number of participants with missing data for each variable of interest	5–6
Outcome data	15 *	Report numbers of outcome events or summary measures	5–6
Main results	16	(*a*) Give unadjusted estimates and, if applicable, confounder-adjusted estimates and their precision (e.g., 95% confidence interval). Make clear which confounders were adjusted for and why they were included	6–9
		(*b*) Report category boundaries when continuous variables were categorized	9–12
		(*c*) If relevant, consider translating estimates of relative risk into absolute risk for a meaningful time period	-
Other analyses	17	Report other analyses done—e.g., analyses of subgroups and interactions, and sensitivity analyses	-
**Discussion**			
Key results	18	Summarize key results with reference to study objectives	12–13
Limitations	19	Discuss limitations of the study, taking into account sources of potential bias or imprecision. Discuss both direction and magnitude of any potential bias	15–16
Interpretation	20	Give a cautious overall interpretation of results considering objectives, limitations, multiplicity of analyses, results from similar studies, and other relevant evidence	13–15
Generalizability	21	Discuss the generalizability (external validity) of the study results	15
**Other information**			
Funding	22	Give the source of funding and the role of the funders for the present study and, if applicable, for the original study on which the present article is based	-

* Give information separately for cases and controls in case-control studies and, if applicable, for exposed and unexposed groups in cohort and cross-sectional studies.

**Table A2:** Authors’ translation of the Informed Consent.

Esteemed participant, the present survey has been developed and shared with the aim to assess the knowledge of occupational physicians on the topic of Legionnaires’ disease and its prevention in occupational settings. The present survey has only scientific aims. No economic or similar compensation is guaranteed to the participants.
While we thank you for your cooperation, we stress that web-based surveys must fulfill the requirements represented by the “Helsinki protocol” and EU Regulation 2016/679.
In order to fulfill the requirements of the Helsinki protocol, we’re requesting to formally share your consent. Without your consent, the survey will not continue. Even after your consent, you can leave the present survey at any moment, until the sharing of the questionnaire (button “share module” at the end of the questionnaire. Moreover, we stress that the questionnaire will be registered in anonymous form, and in no way can it be associated with the compiler, as we will not retain any specific, individual information (e.g., signature, personal address, etc.). All requested personal data are generic and functional to the demographic analyses (gender, age, etc.).
According to the EU Regulation 2016/279 (GDPR), we also state that:
(1) The data controller and processor, as well as the person responsible for data retention during the analyses, will be Dr. ********** whom you can ask about the process through his personal email (*********). Collected data are generic, with the SOLE SCIENTIFIC AIMS that have been previously reported. Please be aware that all personal data must be shared with Criminal Law Authorities, without previous personal consent, in the cases that specifically require reporting by the current legal framework, without a specific request; retrieved data will not be shared with third parties. (2) After the completion of the questionnaire, we cannot identify in any way the compiler; as the questionnaire is totally anonymous by design, we cannot perform any modification or correction of data collected as well as their removal. (3) Data will be retained only for the time strictly required for the aforementioned analyses.
DO YOU AGREE IN PARTICIPATING IN THE PRESENT SURVEY? (YES) (NO)

**Table A3:** Authors’ translation of the questionnaire.

**Section 1. Your Personal Experience with LD Infections** **during your clinical practice**	
Have you previously participated in the risk assessment for LD in any of the enterprises you assist as an occupational physician?	[YES] [NO] [NO ANSWER]
Have you previously promoted any preventive measures for LD in any of the enterprises you assist as an occupational physician??	[YES] [NO] [NO ANSWER]
Have you previously managed any case of LD among assisted workers?	[YES] [NO] [NO ANSWER]
To your knowledge, has any case of LD occurred among your friends/relatives?	[YES] [NO] [NO ANSWER]
**Section 2. To your knowledge (please mark the correct answer)**	
Q1. LD typically has inter-human spreading	[TRUE ] [FALSE] [DON’T KNOW]
Q2. Immunocompromised patients are at higher risk for developing LD	[TRUE ] [FALSE] [DON’T KNOW]
Q3. *Legionella pneumophila* is rare in the environment	[TRUE ] [FALSE] [DON’T KNOW]
Q4. *Legionella pneumophila* optimal growth occurs between 32 and 40 °C	[TRUE ] [FALSE] [DON’T KNOW]
Q5. *Legionella pneumophila* replicates between 20 and 50 °C	[TRUE ] [FALSE] [DON’T KNOW]
Q6. *Legionella* strains able to cause Pontiac Fever cause Legionellosis	[TRUE ] [FALSE] [DON’T KNOW]
Q7. Legionellosis is a vaccine-preventable disease	[TRUE ] [FALSE] [DON’T KNOW]
Q8. Incubation for legionellosis ranges between 2 and 10 days	[TRUE ] [FALSE] [DON’T KNOW]
Q9. Macrolides and Quinolones can be used in cases of suspected LD	[TRUE ] [FALSE] [DON’T KNOW]
Q10. LD occurs in less than 5% of all patients exposed to waters contaminated by *Legionella*	
Q11. LD must be officially reported to the Local Health Unit	[TRUE ] [FALSE] [DON’T KNOW]
Q12. LD is a notifiable disease to international authorities	[TRUE ] [FALSE] [DON’T KNOW]
Q13. LD follows ingestion of contaminated water	[TRUE ] [FALSE] [DON’T KNOW]
Q14. Case fatality for Legionellosis is	
< 1%	[ ]
between 1 and 5%	[ ]
between 5 and 10%	[ ]
between 10 and 15%	[ ]
> 15%	[ ]
Q15. Every year … are reported in Italy	
less than 100 cases	[ ]
100 to 200 cases	[ ]
200 to 500 cases	[ ]
500 to 1000 cases	[ ]
over 1000 cases	[ ]
3. According to your current understanding, in occupational settings, LD can be defined	
in terms of its occurrence, as a disease that is	(1) Extremely infrequent (2) Infrequent (3) Neither frequent nor infrequent (4) Frequent (5) Very frequent
in terms of its severity, as a disease that is	(1) Not at all severe (2) Of low severity (3) Neither severe nor indolent (4) Severe (5) Very severe
4. From your current understanding, how would you rate the reliability of the following diagnostic options for LD (1 = totally unsatisfying; 5 = totally satisfying)	
Broncho-alveolar lavage (BAL)	(1)(2)(3)(4)(5)
Urinary Antigen for Legionella	(1)(2)(3)(4)(5)
Chest CT scans	(1)(2)(3)(4)(5)
Chest X ray studies	(1)(2)(3)(4)(5)
Clinical examination	(1)(2)(3)(4)(5)
5. From your current understanding, how would you rate the occurrence of the following clinical features during LD? (1 = never; 2 = rarely; 3 = unsure; 4 = often; 5 = always)	
Fever, < 38 °C	(1)(2)(3)(4)(5)
Fever, 38 to 41 °C	(1)(2)(3)(4)(5)
Headache	(1)(2)(3)(4)(5)
Conjunctival hemorrhage	(1)(2)(3)(4)(5)
Asthenia	(1)(2)(3)(4)(5)
Cough	(1)(2)(3)(4)(5)
Muscle pain	(1)(2)(3)(4)(5)
Dyspnea	(1)(2)(3)(4)(5)
Chest pain	(1)(2)(3)(4)(5)
Abdominal pain	(1)(2)(3)(4)(5)
6. From your current understanding of LD, which one(s) of the following individual factors increase(s) the risk of developing LD?	
Solid organ transplantation	[ ]
Age > 65 years	[ ]
Smoking history	[ ]
Immune deficiency	[ ]
COPD	[ ]
Diabetes	[ ]
Chronic Kidney Disease	[ ]
Alcohol consumption	[ ]
Previous use of steroids	[ ]
Neoplasia (previous diagnosis)	[ ]
7. From your current understanding of LD, which one(s) of the following environmental factors increase(s) the risk of developing LD?	
Home air conditioners	[ ]
Industrial air conditioners	[ ]
Cooling towers	[ ]
Sewage	[ ]
Hospitals	[ ]
Nursing Homes	[ ]
Swimming Pools	[ ]
Spas/Hot springs	[ ]
Irrigation plants	[ ]
8. In the case that an effective vaccine against WNV is made available, what amount would you suggest is as acceptable expense for the general population for a single shot?	
Not interested	[ ]
Free or < 10 €/shot	[ ]
10–19 €/shot	[ ]
20–29 €/shot	[ ]
30–39 €/shot	[ ]
40–49 €/shot	[ ]
50–100 €/shot	[ ]
>100 €/shot	[ ]
9. Please provide some general information about you:	
Year of birth	______________
Year of medical qualification	______________
You identify yourself as	[Male] [Female] [No Answer]
Do you work in hospital settings?	[yes] [no] [no answer]
Do you work as Occupational Physician for any hospital?	[yes] [no] [no answer]
Do you work as Occupational Physician for any nursing home?	[yes] [no] [no answer]
Do you work as Occupational Physician for any wastewater treatment plant?	[yes] [no] [no answer]
